# Distribution of endemic and introduced tick species in Free State Province, South Africa

**DOI:** 10.4102/jsava.v86i1.1255

**Published:** 2015-06-09

**Authors:** Ivan G. Horak, Adri J. Jordaan, Pierre J. Nel, Joseph van Heerden, Heloise Heyne, Ellie M. van Dalen

**Affiliations:** 1 Department of Veterinary Tropical Diseases, University of Pretoria, South Africa; 2 Department of Zoology and Entomology, University of the Free State, South Africa; 3 Clinvet International, Universitas, South Africa; 4 Department of Economic Development, Tourism and Environmental Affairs, Free State Province, South Africa; 5 Kimberley Veterinary Clinic, Kimberley, South Africa; 6 Parasites, Vectors and Vector-borne Diseases Programme, ARC-Onderstepoort Veterinary Institute, South Africa

## Abstract

The distributions of endemic tick vector species as well as the presence of species not endemic to Free State Province, South Africa, were determined during surveys or opportunistic collections from livestock, wildlife and vegetation. Amongst endemic ticks, the presence of *Rhipicephalus appendiculatus* was confirmed in the north of the province, whilst *Rhipicephalus decoloratus* was collected at 31 localities mostly in the centre and east, and *Ixodes rubicundus* at 11 localities in the south, south-west and centre of the province. Amongst the non-endemic species adult *Amblyomma hebraeum* were collected from white rhinoceroses (*Ceratotherium simum*) on four privately owned farms, whilst the adults of *Rhipicephalus microplus* were collected from cattle and a larva from vegetation at four localities in the east of the province. The collection of *Rhipicephalus evertsi mimeticus* from a sheep in the west of the province is the second record of its presence in the Free State, whereas the presence of *Haemaphysalis silacea* on helmeted guineafowl (*Numida meleagris*) and vegetation in the centre of the province represents a first record for this species in the Free State. The first collection of the argasid tick, *Ornithodoros savignyi*, in the Free State was made from a domestic cow and from soil in the west of the province. The localities at which the ticks were collected have been plotted and the ticks’ role in the transmission or cause of disease in domestic livestock and wildlife is discussed.

## Introduction

Cattle and the larger wildlife species are the preferred hosts of the adults of a multitude of ixodid tick species in South Africa. Some of these ticks are important vectors of disease or toxins affecting domestic livestock as well as some naïve wildlife species. The purchase and sale of wildlife is a flourishing industry in South Africa, and despite their high prices, it is particularly the larger species that are sought after. Amongst these are white and black rhinoceroses (*Ceratotherium simum* and *Diceros bicornis*), African buffaloes (*Syncerus caffer*), roan antelopes (*Hippotragus equinus*), sable antelopes (*Hippotragus niger*), and elands (*Tragelaphus oryx*). Moreover, there is also brisk trade in smaller species.

Although all animals should be tick-free or at least treated with an acaricide before transportation, this is not always an adequate precaution. Hence, with their hosts, ticks may be translocated to regions where neither they nor their hosts are endemic. In addition to the ticks they may harbour, cattle and wildlife may also be asymptomatic carriers of tick-borne diseases that do not occur in their new habitat, with consequent severe implications for resident domestic livestock. Conversely, susceptible introduced livestock and wildlife may be exposed to endemic ticks and tick-borne diseases or pathogens in their new habitat.

The four most important indigenous tick species harboured by cattle and wildlife are *Amblyomma hebraeum*, *Rhipicephalus appendiculatus*, *Rhipicephalus decoloratus* and *Ixodes rubicundus*. Cattle are also the preferred hosts of a fifth species, *Rhipicephalus microplus*, not endemic to South Africa, but now well established here. It was purportedly introduced on cattle imported into Madagascar from southern Asia and thence to South Africa more than 100 years ago (Hoogstraal 1956).

*Amblyomma hebraeum* is the vector of *Ehrlichia ruminantium*, the causative organism of heartwater in cattle, sheep, goats and certain wildlife species (Allsopp, Bezuidenhout & Prozesky 2004). Immune cattle and some wildlife may also serve as reservoirs of infection (Allsopp *et al*. 2004). The brown ear tick, *R. appendiculatus*, is the vector of *Theileria parva*, the causative organism of East Coast fever in cattle and also the vector of a buffalo-derived strain of *T. parva*, responsible for Corridor disease in cattle (Lawrence, Perry & Williamson 2004). The one-host tick, *R. decoloratus*, transmits *Babesia bigemina*, the causative organism of African redwater in cattle (De Vos, De Waal & Jackson 2004), whilst African buffaloes may serve as asymptomatic carriers of this organism (De Vos *et al*. 2004). The aggressively invasive *R. microplus* transmits *B. bigemina* as well as the more virulent *Babesia bovis*, the causative organism of Asiatic redwater in cattle (De Vos *et al*. 2004). In addition, both *R. decoloratus* and *R. microplus* can transstadially or intrastadially transmit *Anaplasma marginale*, the causative organism of anaplasmosis or gall sickness in cattle (Potgieter 1981).

Although cattle appear to be the preferred domestic hosts of the Karoo paralysis tick, *I. rubicundus* (Fourie, Kok & Heyne 1996), it is sheep that become paralysed and thousands may die annually (Spickett & Heyne 1988). Regarding two lesser-known ixodid ticks, *Rhipicephalus evertsi mimeticus*, primarily a Namibian tick introduced into the Free State, is a potential cause of paralysis in lambs and calves (Gothe, Gold & Bezuidenhout 1986), whilst *Haemaphysalis silacea*, whose distribution is largely confined to the Valley Bushveld of the Eastern Cape Province, appears to cause no harm. The argasid tick, *Ornithodoros savignyi*, colloquially known as the Kalahari sand tampan, when feeding in by large numbers may lead to anaemia in young calves or symptoms similar to those of anaphylactic shock in slightly older animals. Paralysis associated with the feeding of this tick has also been reported (Spickett 1984).

On a 1978 map illustrating the geographic distribution of *R. appendiculatus* in South Africa, its presence in the Free State is indicated by means of question marks at two localities in the north of the province (Howell, Walker & Nevill 1978). More recently, however, consistent confirmed collections of this species have been reported in this region (Spickett 2013). Although *R. appendiculatus* was supposedly collected recently at two localities in the centre of the Free State and one in the south (Tonetti *et al*. 2009), these ticks were subsequently identified by the first author as *Rhipicephalus warburtoni*, and an erratum was published in a subsequent edition of the relevant journal. *Rhipicephalus decoloratus* is widespread in the eastern half of the Free State, with pockets in the north-west, south-west and south of the province (Howell *et al*. 1978; Spickett 2013). The distribution of *I. rubicundus* includes the southern, south-western and south-eastern regions of the Free State (Howell *et al*. 1978; Spickett & Heyne 1988). With the exception of a larva and a nymph collected from sheep more than 30 years ago on a farm in the north-eastern region of the province (Horak, Williams & Van Schalkwyk 1991b), *A. hebraeum* is not present in the Free State. There is a single record of *R. microplus* in the north-east of the province and another in the north-west (Tonetti *et al*. 2009), and a single record of *R. evertsi mimeticus* in the south-west of the province (Fourie, Horak & Marais 1988). Neither *H. silacea* nor *O. savignyi* have previously been reported in the Free State.

The present article combines the results of surveys aimed at determining the hosts, species composition, geographic distributions and seasonal abundance of ticks in the Free State with those obtained from opportunistic collections of ticks in the same province. It highlights the tick species and the potential tick-borne diseases introduced into the Free State on domestic livestock and wildlife as well as the occurrence of pre-existing diseases in non-endemic wildlife species in the province.

## Research method and design

Three long-term surveys, from which some of the present results were derived, were conducted in the Free State. In the first survey, ticks were collected from domestic and wild animals and by dragging flannel strips over vegetation at several localities spread throughout the province (Jordaan 2011). The second survey focused on determining the presence of *R. appendiculatus* on African buffaloes and on commercial cattle farms adjacent to buffalo ranches (Jordaan 2011). The third survey was aimed at determining the geographic distribution of *I. rubicundus* and to this end sheep were examined for adult ticks and eastern rock elephant shrews (*Elephantulus myurus*) for immature ticks at a total of 11 localities (Horak & Fourie 1992; Fourie, Horak & Woodall 2005). Ixodid ticks were also collected opportunistically from chemically immobilised white rhinoceroses on four wildlife ranches and from a paralysed sheep in the west of the province. Argasid ticks were collected opportunistically from a paralysed domestic cow and the soil surface in the west of the province.

Tick collections were made from cattle whilst they were restrained in a crush and from sheep restrained by the operator's assistants. Elephant shrews were trapped and ticks collected from them before they were released, or they were euthanised and ticks collected from their carcasses, whilst helmeted guineafowl (*Numida meleagris*) and some of the larger wildlife species listed in the tables as being from elsewhere had been shot on farms or in reserves and their carcasses processed for tick recovery. The tick burdens of the latter animals have all previously been published by Fourie and Horak and their co-workers (Horak *et al*. 1991a; Fourie, Horak & Van den Heever 1992; Horak, Golezardy & Uys 2007), and have been included in the present article for comparative purposes. The rhinoceroses and buffaloes from which ticks were collected in the present study had been immobilised for other management-related procedures.

All the ticks collected were identified and counted under a stereoscopic microscope and the localities at which they had been collected were plotted. For security reasons the actual localities at which the rhinoceroses were immobilised were not plotted but instead those of the nearest towns. Two of the authors each have more than 30 years of experience in the identification of the adult stages of ticks that occur in South Africa and hence there was no necessity to consult other sources as an aid towards identification.

**FIGURE 1:**
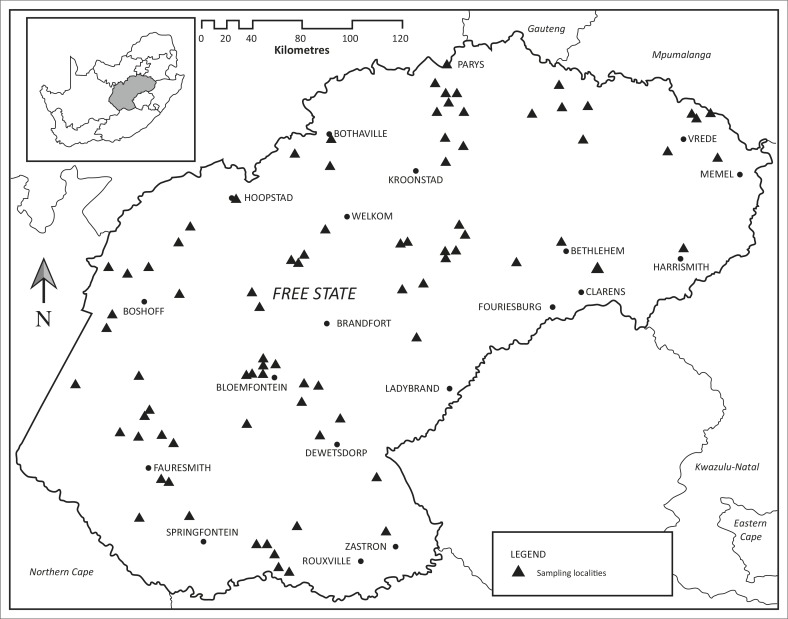
Sampling localities at which ticks were collected in Free State Province, South Africa.

**FIGURE 2:**
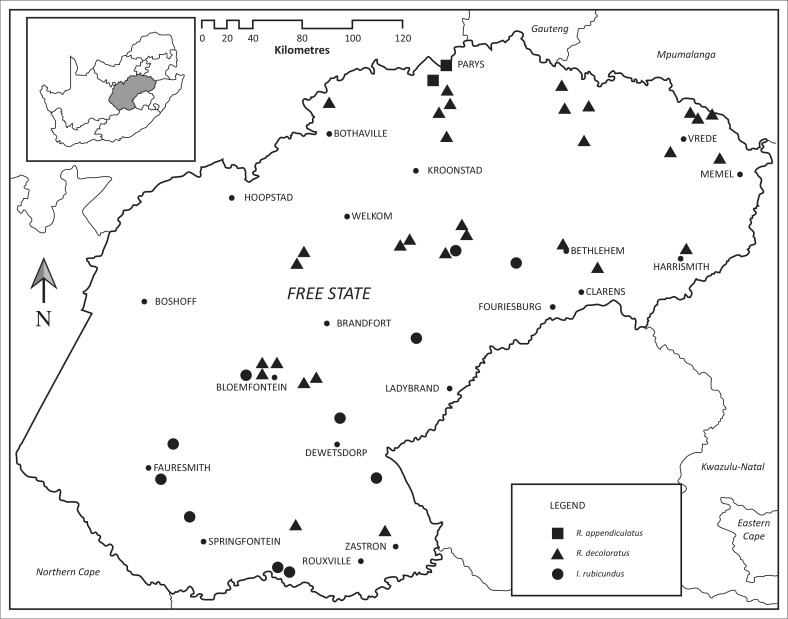
Localities at which the ticks *Rhipicephalus appendiculatus*, *Rhipicephalus decoloratus* and *Ixodes rubicundus* were collected in Free State Province, South Africa.

**FIGURE 3:**
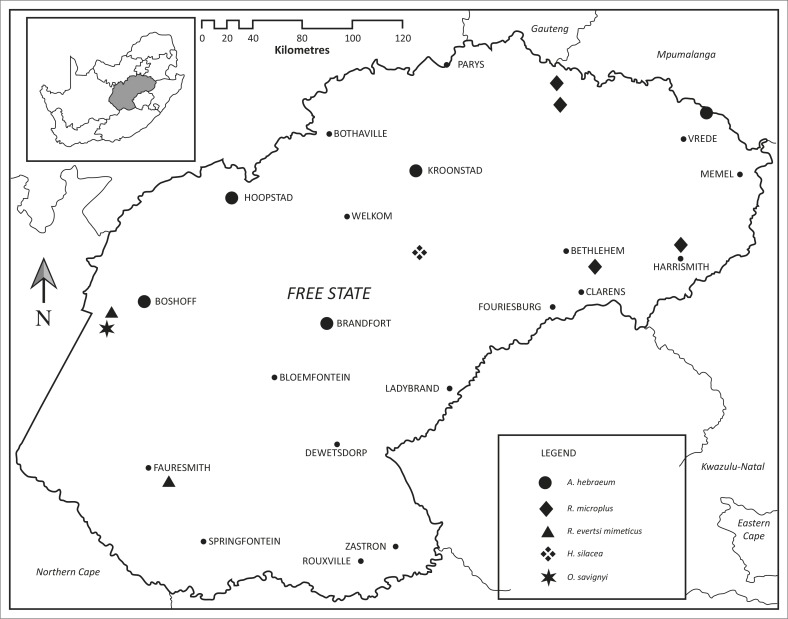
Localities at which the ticks *Amblyomma hebraeum*, *Rhipicephalus microplus*,* Rhipicephalus evertsi mimeticus*, *Haemaphysalis silacea* and *Ornithodoros savignyi* were collected in Free State Province, South Africa.

The localities at which ticks were collected in the three long-term surveys as well as those at which ticks were collected opportunistically are plotted in Figure 1, whilst the localities at which the eight tick species pertinent to this study were collected are plotted in Figures 2 and 3. The numbers of *R. appendiculatus*, *R. decoloratus* and *I. rubicundus* collected from host animals, and where applicable from vegetation, in the Free State and from the same host species and vegetation within the core distributions of these ticks elsewhere in South Africa have been summarised in Table 1, whilst the same has been done for *A. hebraeum*, *R. microplus*, *R. evertsi mimeticus*, *H. silacea* and *O. savignyi* in Table 2.

**TABLE 1:** Major tick species endemic to Free State Province, South Africa and their hosts.

Tick species	Origin	Hosts or vegetation	Number of collections	Number of ticks collected			
				Larvae	Nymphs	Males	Females
*Rhipicephalus appendiculatus*	Free State	Cattle†	8	5	7	26	17
		Buffaloes†	19	1	54	5	0
		Vegetation†	29	265	51	3	1
	Elsewhere	Cattle‡	109	194	39	31	17
		Buffaloes‡	18	7744	1818	76	43
		Vegetation‡	527	97	3	< 0.4	< 0.6
*Rhipicephalus decoloratus*	Free State	Cattle†	55	0	20	52	202
		Buffaloes†	7	0	4	2	10
		Vegetation†	120	1552	0	0	0
	Elsewhere	Cattle‡	298	0.7	0.8	0.9	3
		Buffaloes‡	11	149	26	23	15
		Vegetation‡	1063	94	< 0.1	< 0.1	0
*Ixodes rubicundus*	Free State	Sheep‡	46	0	0	< 0.4	3.8
		Elephant shrews‡	99	15	11	0	0
	Elsewhere and Free State	Sheep‡	816	0	0	3	6
		Elephant shrews‡	137	32	19	0	0

†, Total number of ticks; ‡, Mean number of ticks.

**TABLE 2:** Non-endemic tick species collected in Free State Province, South Africa and their hosts.

Tick species	Origin	Hosts or vegetation	Number of collections	Number of ticks collected	
				Larvae	Nymphs	Males	Females
*Amblyomma hebraeum*	Free State	White rhinoceroses†	8	0	0	15	12
	Elsewhere	White rhinoceroses‡	6	24	74	180	106
		Black rhinoceroses‡	14	21	29	76	34
*Rhipicephalus microplus*	Free State	Cattle†	4	0	0	2	7
		Vegetation†	1	1	0	0	0
	Elsewhere and Free State	Cattle‡	608	0	0.1	2	5
		Vegetation‡	553	11	0	< 0.1	< 0.1
		Eland†	1	0	0	32	48
		Gemsbok†	3	0	0	0	8
		Grey rhebok†	1	0	0	2	8
*Rhipicephalus e. mimeticus *	Free State	Sheep†	1	0	0	3	1
	Namibia	Mountain zebras‡	12	158	169	29	9
*Haemaphysalis silacea*	Free State	Guineafowl†	2	192	1	0	0
		Vegetation†	2	15	0	0	0
	Elsewhere	Guineafowl‡	89	87	17	0	0
		Vegetation‡	90	33	< 1.0	0	< 0.1
*Ornithodoros savignyi*	Free State	Domestic cow†	1	0	6	2	1
		CO_2_ trap†	1	0	508	49	16

†, Total number of ticks; ‡, Mean number of ticks.

## Ethical considerations

Tick collections made from cattle and sheep were performed with the consent of the approximately 50 owners of these animals. Ticks were also collected from a paralysed sheep as well as from a paralysed cow. Rhinoceroses and buffaloes from which ticks were collected in the present study had been chemically immobilised by a veterinarian for other management-related procedures with the consent of their owners, and ticks were collected opportunistically from these animals. Rock elephant shrews were trapped and released once their ticks had been collected, or euthanised and ticks were collected from their carcasses, whilst guineafowl and some of the larger wildlife species listed in the tables as being from elsewhere had been shot on farms or in reserves and their carcasses processed for tick recovery. The elephant shrews, guineafowl and larger wildlife species were examined in the 1980s or 1990s with the consent of the National Parks Board of Trustees, the Division of Nature Conservation of the Cape Provincial Administration and the Natal Parks Board. The tick burdens of these animals have all previously been published by Fourie and Horak and their co-workers (Horak *et al*. 1991a, 2007; Fourie *et al*. 1992) and have been included in the present article for comparative purposes.

## Results and discussion

### Endemic species

#### Rhipicephalus appendiculatus

A single larva, some nymphs and a male tick were collected from buffaloes on a farm in tree and bush savannah close to Parys in the northern Free State, and larvae and nymphs from drag-samples of vegetation on the same farm (Figure 2). Moreover, larvae, nymphs and adult ticks were collected from cattle and from vegetation on two commercial farms adjacent to the buffalo ranch. The collection of *R. appendiculatus* in the north of the province confirms its presence there, a fact that was previously only speculative (Howell *et al*. 1978), but confirmed by subsequent collections (Spickett 2013). Its recovery from cattle, buffaloes and vegetation indicate that it is well established in this habitat. This set of circumstances creates the ideal environment for the transmission of buffalo-derived *T. parva* from buffaloes to cattle, with the resultant occurrence of Corridor disease and mortality in the cattle, should an infected buffalo be introduced into this region of the Free State. Moreover, the spread of the organism from an infected buffalo to ‘disease-free’ buffaloes can have considerable financial implications as a result of the huge decrease in value of infected buffaloes and the attendant quarantine restrictions instituted by veterinary authorities.

All stages of development of *R. appendiculatus* prefer large animals as hosts and all stages quest for hosts from vegetation. Elsewhere considerable numbers have been collected from cattle, buffaloes and vegetation (Table 1).

#### Rhipicephalus decoloratus

Nymphs and adults were collected from cattle and buffaloes, and larvae from drag-samples of vegetation at a total of 31 localities, of which two were west of Bloemfontein and two in the south-east of the province (Figure 2). Elsewhere large numbers of all stages of development of this one-host tick have been collected from buffaloes and vegetation (Horak *et al*. 2007; Horak, Gallivan & Spickett 2011) (Table 1). Most cattle within the distribution range of *R. decoloratus* are infested, but the numbers of ticks they may harbour are usually low because of the regular application of acaricides.

*Rhipicephalus decoloratus* is endemic to the eastern grasslands of the Free State, and in the present study it was collected east of longitude 26° E, which bisects the province slightly to the west of Bloemfontein (Figure 2). Its spread westwards is closely related to the 500 mm mean annual precipitation isohyet, which follows a north-south line that passes through the Bloemfontein region. Few resident cattle within the tick's region of endemism suffer from African redwater caused by *B. bigemina* transmitted by *R. decoloratus*. Most are infected when they are calves and are protected by the passive transfer of immunity via colostrum from their immune dams during the first 2 months of their lives, or by an innate, non-specific resistance until they are 6 months of age (De Vos *et al*. 2004). This immunity, which is not necessarily absolute, may be life-long and is independent of re-infection. The stress of calving or of disease may lead to a breakdown in immunity and subsequent incidents of babesiosis. Susceptible adult cattle introduced into a region where infection is prevalent are likely to suffer severe morbidity and mortality. African buffaloes can develop latent infections and serve as a source of infection for cattle (De Vos *et al*. 2004).

#### Ixodes rubicundus

Adult *I. rubicundus* were collected from sheep in the south and north-east of the Free State, and larvae and nymphs from rock elephant shrews in the south-west and centre of the province (Figure 2). Burdens of adult ticks are never very large and sheep and cattle are amongst the preferred hosts of this stage of development, whilst the immature stages by preference infest rock elephant shrews (Horak & Fourie 1992; Fourie *et al.* 1996, 2005) (Table 1).

With the exception of caracals (*Caracal caracal*), which are infested by all stages of development, *I. rubicundus* requires two completely differently sets of hosts to complete its life cycle. The adults infest wild ruminants that inhabit rocky outcrops, hills and mountain slopes or visit these localities for browsing, whilst the larvae and nymphs are strictly parasites of elephant shrews and red rock rabbits (*Pronolagus* spp.). Thousands of sheep may die annually from paralysis induced by the saliva of feeding female ticks (Spickett & Heyne 1988). *Ixodes rubicundus* has also caused paralysis and mortality amongst introduced gemsbok (*Oryx gazella*) in the south of the province (Fourie & Vrahimis 1989), as well as in introduced roan and sable antelopes (J. van Heerden [Kimberley Veterinary Clinic], pers. comm., 10 May 2014). Paralysis and mortality in springbok (*Antidorcas marsupialis*) have also been reported (Fourie & Horak 1987). This seems to occur when farmers move their sheep to grassveld plains during winter, causing springbok to migrate from there to mountainous terrain, the preferred habitat of the tick.

### Non-endemic species

#### Amblyomma hebraeum

Seven male and three female ticks were collected off two rhinoceroses from the Kruger National Park, four days after they had been released on a farm near Kroonstad during June 2012, and a male and a female tick in November 2013, off a rhinoceros from Gauteng 13 days after it had been released (Figure 3). A single male tick was collected from a rhinoceros at Boshof during August 2013, whereas the last rhinoceroses released there apparently came from the Kruger National Park in 2010. At Brandfort, two males and five female ticks were collected off a rhinoceros translocated from Limpopo Province on the day it was released in February 2013, two male ticks from another animal during April 2013, nearly 2 months after it had been introduced from North West Province and a male and female tick during July 2013 off a third animal from Gauteng Province 11 days after it had been released. At Hoopstad, a single male tick was collected during November 2012 off a rhinoceros from Limpopo 6 weeks after it had been released. The male ticks collected from the rhinoceros near Brandfort nearly 2 months after it had been introduced from North West Province and from the rhinoceros near Hoopstad 6 weeks after it had been translocated from Limpopo Province are not necessarily an indication of recently acquired infestations, as male ticks can remain attached for up to 8 months (Jordaan & Baker 1981).

Although a larva and a nymph of *A. hebraeum* had been collected 30 years previously from sheep in the Vrede region of the north-eastern Free State (Horak *et al.* 1991b), no subsequent collections have been made there. The recovery now of adult *A. hebraeum* from rhinoceroses at four localities in the Free State constitutes new distribution records, with the ticks undoubtedly introduced into the province on these animals. Whether *A. hebraeum* has become established at these localities remains to be seen. It is possible that it has done so near Boshof, where a single male tick was collected from a rhinoceros during 2013, 3 years after the last rhinoceroses had been introduced there from the Kruger National Park. The apparently frequent introduction of rhinoceroses, and with them *A. hebraeum*, at Kroonstad and Brandfort could over time perhaps lead to the establishment of viable populations there.

The immature stages of *A. hebraeum* infest a large variety of hosts, including mammals, birds and reptiles, whilst the adults by preference infest truly large animals(Horak *et al*. 1987). Elsewhere in South Africa fairly large numbers of all stages of development have been recovered from white and black rhinoceroses (Table 2).

Should *A. hebraeum* become established in the Free State in regions in which the climate and vegetation are suitable for its survival, it will be nearly impossible to eradicate because of the approximately 15 000 eggs laid by engorged females, the subsequently large numbers of larvae that have hatched and then quest for hosts from vegetation, and the variety of large and small wildlife hosts that they infest. The establishment of viable populations of *A. hebraeum* in the Free State will have serious implications for the transmission of heartwater to susceptible animals in the province, where to date no cases of this disease have been positively diagnosed. However, should cattle or antelope species that are asymptomatic carriers of the organism be translocated from regions in which heartwater is endemic to properties in the Free State where *A. hebraeum* has become established, this could give rise to foci of infected ticks. These in turn could transmit infection to susceptible cattle, sheep and goats and wildlife with resultant high morbidity and mortality.

#### Rhipicephalus microplus

Two male and seven female *R. microplus* were collected from four cattle and a single larva from a drag-sample at a total of four localities, two in the north-east and two in the east of the Free State (Figure 3). This is the third record for this tick in the province, the first being that from gemsbok in the north-west of the province (Tonetti *et al*. 2009) and the second from cattle in the north-east of the province (Spickett 2013). *R. microplus* has been collected from a large number of cattle and also from an eland, three gemsbok (*Oryx gazella*) and a grey rhebok (*Pelea capreolus*) (Table 2). It is now present in most habitats favoured by *R. decoloratus* in South Africa and is displacing it in several of these (Howell *et al*. 1978; Tønnesen *et al*. 2004; Horak *et al*. 2009; Nyangiwe, Harrison & Horak 2013). It was thought to be strictly a parasite of cattle, but it would seem that it is adapting to feeding on goats and wildlife (Horak *et al*. 2009; Tonetti *et al*. 2009). Indeed, the first record of its presence in the Free State is on gemsbok (Tonetti *et al*. 2009), whilst fairly large numbers of adult ticks have been collected from an eland in the Western Cape Province that had no recent contact with cattle (Table 2). Its introduction into the eastern region of the Free State probably took place on infested cattle. Cattle are moved by some farmers between the Free State and KwaZulu-Natal for better pastures, and infestation could be acquired there and transferred in this way.

*Rhipicephalus microplus* transmits both *B. bigemina* and the more virulent *B. bovis* (De Vos *et al*. 2004). Should cattle or ticks infected with *B. bovis* be brought into the Free State, severe cases of Asiatic redwater and mortality are likely to occur in susceptible resident animals. Where *B. bovis* is suspected as the cause of disease it is imperative that a definite diagnosis be made, and also advisable that ticks for identification be collected from the diseased animal and/or other cattle on the farm in order to institute precautionary measures against future outbreaks.

#### Rhipicephalus evertsi mimeticus

There is a 1988 record of *R. evertsi mimeticus* in the south-west of the Free State (Fourie *et al.* 1988), but no ticks have been collected since then. The opportunistic collection of three male and one female tick from sheep in the west of the province during April 2014 is thus also likely to represent a new introduction and not an established population (Figure 3).

*Rhipicephalus evertsi mimeticus* is indigenous to Namibia and Angola, but has been introduced into South Africa on more than one occasion, and it was then speculated as to whether it would become established here (Walker, Keirans & Horak 2000). The collection of *R. evertsi mimeticus* at nine localities in North West Province indicates that this has indeed occurred (Spickett, Heyne & Williams 2011). Although there are no field reports incriminating *R. evertsi mimeticus* as the cause of paralysis in sheep, it has been demonstrated experimentally (Gothe *et al.* 1986). The sheep from which it was collected now was one of a group of five sheep exhibiting paresis or paralysis (J. van Heerden [Kimberley Veterinary Clinic], pers. comm., 10 May 2014). These sheep were also infested with *R. evertsi evertsi*. Both ticks have a preference for equids as hosts and 12 mountain zebras, *Equus zebra*, examined in the Khomas Hochland of Namibia, were all infested and fairly large numbers of all stages of development of *R. evertsi mimeticus* were recovered (Horak, Biggs & Reinecke 1984) (Table 2).

#### Haemaphysalis silacea

Larvae and a nymph of *H. silacea* were collected from helmeted guineafowl and larvae from vegetation in the Willem Pretorius Nature Reserve in the central Free State (Jordaan 2011) (Figure 3). This is the first record of this species in the province. All parasitic stages of development of *H. silacea* infest tragelaphine antelope species as well as other wild bovids and domestic livestock in the Albany thickets biome of the Eastern Cape Province. Large numbers of larvae and nymphs have also been collected from helmeted guineafowl (Horak *et al*. 1991a). *H. silacea* was probably introduced into the Willem Pretorius Nature Reserve in the Free State on buffaloes or elands translocated from the Eastern Cape. It has now become established in this reserve as evidenced by the collection of the immature stages from guineafowl and larvae from vegetation, indicating that a substantial population of adult ticks is present on larger hosts. This tick is believed to transmit no tick-borne pathogens.

#### Ornithodoros savignyi

Two male ticks, a single female tick and six nymphs were collected from a paralysed domestic cow on a farm approximately 10 km west of Bothasput in the western Free State (Figure 3), whilst 49 males, 16 females and 508 nymphs were collected from soil on the same farm by means of a carbon dioxide trap (J. van Heerden [Kimberley Veterinary Clinic], pers. comm., 10 May 2014) (Table 2). This is the first record of *O. savignyi* in the Free State.

The argasid tick *O. savignyi*, colloquially known as the Kalahari sand tampan, is present in the arid and semi-arid sandy regions of the Northern Cape Province and Namibia (Theiler 1962). It is usually found in sandy soil in the shade of trees and its nymphs and adults feed on the larger species of wildlife and on domestic livestock resting under these trees. It secretes a toxin with its saliva, which has been reported to cause paralysis and mortality in its hosts (Spickett 1984). Several African buffaloes, sable antelopes and domestic cattle had become paralysed and had died on the farm on which *O. savignyi* was collected for the first time in the Free State, presumably from a toxin secreted with the saliva of the ticks. Buffaloes, sable antelopes and cattle seek the shade of trees in the heat of the day, thus being exposed to infestation, whilst roan antelopes on the farm were seemingly unaffected as they prefer open savannah and are less likely to make use of the shade.

The closest record of *O. savignyi* to the Free State is to the east of Hopetown in the eastern region of the Northern Cape Province (Theiler 1962). Judging by the large number of ticks collected by means of a dry ice trap, it now appears to be well established in the Free State west of Bothasput. Even though *O. savignyi* only feeds for short periods, some ticks remain on hosts for longer. It is these ticks, which were probably introduced into the Free State on domestic livestock purchased within the tick's core distribution range, which have established a foothold in the province. Once established, it is virtually impossible to get rid of sand tampans. All stages live under the soil surface and only emerge to feed. Several months may elapse between blood-meals and the ticks are capable of surviving for years.

## Conclusion

The four major tick vectors of disease to cattle have now all been collected in the Free State, namely *A. hebraeum*, *R. appendiculatus*, *R. decoloratus* and *R. microplus*. The presence of *A. hebraeum* and *R. microplus* is at present tenuous and only future surveys will confirm whether they have become established there or not, whereas *R. appendiculatus* and *R. decoloratus* are both well established. The Karoo paralysis tick, *I. rubicundus*, the cause of paralysis in sheep, is also well established in the south and west of the province. The recovery of *H. silacea* and *O. savignyi* are first records for the Free State.
